# Effects of frontal-executive dysfunction on self-perceived hearing handicap in the elderly with mild cognitive impairment

**DOI:** 10.1371/journal.pone.0210014

**Published:** 2019-03-06

**Authors:** Soo Jung Lee, HyangHee Kim, Lee-Suk Kim, Ji-Hye Kim, Kyung Won Park

**Affiliations:** 1 Graduate Program in Speech and Language Pathology, Yonsei University, Seoul, Korea; 2 Department and Research Institute of Rehabilitation Medicine, Yonsei University College of Medicine, Seoul, Korea; 3 Department of Otolaryngology, Head and Neck Surgery, Dong-A University College of Medicine, Busan, Korea; 4 Sorion ENT Clinic, Busan, Korea; 5 Department of Neurology, Dong-A University College of Medicine, Busan, Korea; 6 Institute of Convergence Bio-Health, Dong-A University, Busan, Korea; Universita degli Studi Europea di Roma, ITALY

## Abstract

It is increasingly agreed upon that cognitive and audiological factors are associated with self-perceived hearing handicap in old adults. This study aimed to compare self-perceived hearing handicap among mild cognitive impairment (MCI) subgroups and a cognitively normal elderly (CNE) group and determine which factors (i.e., demographic, audiometric, or neuropsychological factors) are correlated with self-perceived hearing handicap in each group. A total of 46 MCI patients and 39 hearing threshold-matched CNE subjects participated in this study, and their age ranged from 55 to 80 years. The MCI patients were reclassified into two groups: 16 with frontal-executive dysfunction (FED) and 30 without FED. All subjects underwent audiometric, neuropsychological, and self-perceived hearing handicap assessments. The Korean version of the Hearing Handicap Inventory for the Elderly (K-HHIE) was administered to obtain the hearing handicap scores for each subject. After controlling for age, years of education, and depression levels, we found no significant differences in the K-HHIE scores between the MCI and the CNE groups. However, after we classified the MCI patients into the MCI with FED and MCI without FED groups, the MCI with FED group scored significantly higher than did both the MCI without FED and the CNE groups. In addition, after controlling for depression levels, significant partial correlations of hearing handicap scores with frontal-executive function scores and speech-in-noise perception performance were found in the MCI groups. In the CNE group, the hearing handicap scores were related to peripheral hearing sensitivity and years of education. In summary, MCI patients with FED are more likely to experience everyday hearing handicap than those without FED and cognitively normal old adults. Although educational level and peripheral hearing function are related to self-perceived hearing handicap in cognitively normal old adults, speech-in-noise perception and frontal-executive function are mainly associated with hearing handicap in patients with MCI.

## Introduction

Hearing impairment does not necessarily parallel self-perception of hearing handicap in everyday life [1]. According to the World Health Organization, hearing handicap refers to the non-auditory consequences, such as emotional distress and restrictions on social engagement, for an individual due to hearing impairment [2]. Individuals with a similar degree of hearing impairment may or may not experience significant self-perceived hearing handicap in their daily lives because hearing impairment can cause different emotional and social impacts on each individual [3]. Therefore, hearing handicap is a complex phenomenon that involves far more than hearing impairment alone.

Both audiological and non-audiological variables are associated with self-perceived hearing handicap. Several studies indicate an association between self-perceived hearing handicap and patient outcomes on speech-in-noise measures [4–8]. A recent study suggests that self-perceived hearing handicap correlates more highly with speech-in-noise perception outcomes than with general health-related quality of life [9]. Moreover, numerous studies have shown that peripheral hearing sensitivity is correlated with self-perceived hearing handicap in old adults, but the correlations are only weak-to-moderate [1,10]. Audiometric measures explain less than 50% of the variance in self-perception of hearing handicap [11].

The aforementioned studies suggest that non-audiometric factors may play an important role in self-perceived hearing handicap in old adults. Thus, factors other than the degree of hearing impairment may contribute to self-perceived hearing handicap in old adults. One recent study on the impact of cognitive function on self-perceived hearing handicap indicates that old adults with high working memory capacity have more hearing difficulties in their daily lives, and the results contradict the authors’ initial hypothesis [12]. Accordingly, the authors reasoned that old adults with high working memory may have a more active lifestyle, therefore a large reliance on their hearing abilities, and may also be more likely to identify their difficulties. However, because the participants in the aforementioned study were not screened for cognitive disorders, this study did not rule out the possibility that some participants may have cognitive disorders. We speculate that cognitive factors may affect self-perceived hearing handicap differently in cognitively normal old adults and in cognitively impaired old patients.

To overcome such limitations, this study was carefully designed in order to account for the effect of cognitive function on hearing-related difficulties. We hypothesized that old patients with mild cognitive impairment (MCI) would have more hearing-related difficulties than their hearing threshold-matched peers with normal cognitive function would. Specifically, we hypothesized that MCI patients with frontal-executive dysfunction (FED) would report greater hearing handicap than those without FED. Because previous studies have demonstrated that frontal-executive function strongly impacts speech-in-noise perception in patients with MCI [13,14] and speech-in-noise perception performance is highly correlated with self-perceived hearing handicap [4–9], we speculated that MCI patients with FED would have more hearing-related difficulties than those without FED. To the best of our knowledge, no previous studies have focused on self-perceived hearing handicap in patients with MCI. The purpose of the current study was to compare self-perceived hearing handicap among MCI subgroups and a cognitively normal elderly (CNE) group and to determine which demographic, audiometric, and neuropsychological factors are correlated with self-perceived hearing handicap in each group.

## Materials and methods

### Participants

We recruited 46 MCI patients, aged 55 to 80 years, at the Memory and Dementia Clinic at the Dong-A University Medical Center. MCI was diagnosed according to the Petersen’s criteria [15]: 1) subjective memory complaints reported by patients or informants; 2) objective cognitive impairments as defined by age- and education-adjusted scores below -1.0 standard deviations (SD) for at least one test in the neuropsychological battery; 3) without dementia according to the Diagnostic and Statistical Manual of Mental Disorders-4^th^ edition; and 4) normal activities of daily living. Exclusion criteria included a history of major medical, neurological, or psychiatric illness. The MCI patients were further classified into patients with FED (n = 16; 6 men and 10 women) and patients without FED (n = 30; 6 men and 24 women). The FED patients had to meet the following criteria: 1) age- and education-adjusted scores below -1.0 SD on the animal naming or phonemic-letter naming tests in the Controlled Oral Word Association Test (COWAT) [16] and 2) age- and education-adjusted scores below -1.0 SD on the color reading in the Korean version of the Color Word Stroop Test (K-CWST) [17]. If patients did not meet these criteria, they were classified as patients without FED.

We recruited 39 cognitively normal old adults (14 men and 25 women) as a control group. The cognitively normal old adults had to meet the following criteria: 1) no significant underlying medical, neurological, or psychiatric illness; 2) normal performance as defined by age- and education-adjusted scores above -1.0 SD on the Korean version of the Mini-Mental State Examination (K-MMSE) [18], digit span (forward and backward) [19], Seoul Verbal Learning Test (SVLT) (immediate and delayed recall) [20], and COWAT (animal and three-phonemic-letter naming); and 3) no subjective memory complaints.

For hearing acuity, all subjects had to meet the following inclusion criteria: 1) no conductive components on tympanometry and pure tone audiometry; 2) hearing threshold levels at 0.5, 1, and 2 kHz ≤ 25 dB HL; 4 kHz ≤ 40 dB HL; and 8 kHz ≤ 70 dB HL for each ear; 3) no greater than 10 dB HL of inter-aural asymmetry on pure-tone averages (PTAs) (average of 0.5, 1, 2, and 4 kHz); 4) a speech discrimination score ≥ 80% for each ear; and 5) no previous use of hearing aids. The elderly subjects were recruited at a relatively early stage of age-related hearing impairment, which is characterized by threshold elevation beginning at high frequencies. Table 1 shows the demographic data and audiometric test results for each group.

**Table 1:** Demographic data of patients and audiometric test results in each group.

	MCI		CNE(n = 39)	*p*^†^
	MCI with FED (n = 16)	MCI without FED (n = 30)		
Demographics				
Age (years)	66.56 ± 6.12	68.56 ± 6.34	63.92 ± 4.84	0.008**
Men/women	6/10	6/24	14/25	0.296
Education (years)	7.00 (6.00−11.25)	7.50 (6.00−12.00)	12.00 (9.00−14.00)	0.001**
K-MMSE	25.00 (24.00−26.00)	26.00 (24.75−29.00)	29.00 (28.00−30.00)	< 0.001***
SGDS	4.00 (2.25−11.00)	3.50 (2.00−8.00)	1.00 (0.00−2.00)	< 0.001***
Audiometric test results^‡^				
PTAs	21.35 ± 5.23	18.52 ± 5.35	18.72 ± 4.52	0.079
0.25 kHz	17.34 ± 5.35	14.33 ± 6.12	11.60 ± 4.53	0.002**
0.5 kHz	17.18 ± 6.94	14.25 ± 7.25	15.06 ± 4.07	0.282
1 kHz	21.25 (15.62−22.50)	17.50 (12.50−22.50)	17.50 (12.50−22.50)	0.464
2 kHz	25.00 (22.50−25.00)	21.25 (12.50−22.50)	20.00 (15.00−22.50)	0.019*
4 kHz	35.00 (25.00−37.50)	25.00 (17.50−35.62)	22.50 (22.50−35.00)	0.146
8 kHz	50.78 ± 15.10	48.83 ± 21.63	40.00 ± 16.79	0.066
SRT	22.50 (18.12−25.00)	20.00 (15.00−22.50)	20.00 (15.00−22.50)	0.138
MCL	61.25 (58.12−65.00)	60.00 (55.00−62.50)	60.00 (55.00−62.50)	0.243
SDS	98.00 (92.00−100.00)	96.00 (92.00−100.00)	100.00 (96.00−100.00)	0.085
Speech-in-noise perception				
SSN condition	34.68 ± 9.43	41.50 ± 19.40	56.02 ± 16.02	< 0.001***
MBN condition	16.40 ± 13.13	25.83 ± 16.60	53.02 ± 15.17	< 0.001***

Parametric and nonparametric data are presented as mean ± standard deviation and median (interquartile range), respectively.

*P < 0.05,

**P < 0.01,

***P < 0.001.

^†^Parametric and nonparametric data were analyzed using one-way analysis of variance and the Kruskal-Wallis test, respectively.

^‡^All audiometric test results were expressed as dB HL except for the SDS and speech perception results expressed as percentage.

Abbreviations: MCI, mild cognitive impairment; FED, frontal-executive dysfunction; CNE, cognitively normal elderly; K-MMSE, Korean version of Mini-Mental State Examination; SGDS, Short version of Geriatric Depression Scale; PTAs, pure-tone averages; SRT, speech reception threshold; MCL, most comfortable loudness level; SDS, speech discrimination score; SSN, speech-spectrum noise; MBN, multi-talker babble noise.

This study was approved by the Institutional Review Board of the Dong-A University Medical Center, Busan, Korea (IRB#: 16–048). Written informed consent was obtained from every participant before the experimental procedures. The capacity of participants to give informed consent was assessed using the Clinical Dementia Rating (CDR) scale [21]. It has been reported that the CDR could be suggested as a marker of decisional capacity for demented individuals, and individuals with CDR of 0 (no dementia) or 0.5 (very mild dementia or being labeled as mild cognitive impairment) can understand consent information describing a relatively simple and nontreatment research protocol [22]. In the present study, all participants were divided into cognitively normal (CDR = 0) or mild cognitive impairment (CDR = 0.5) subgroups. The clinician determined if the patients have the capacity to give the informed consent.

### Experimental measurements

#### Audiometric assessments

**Fig 1:**
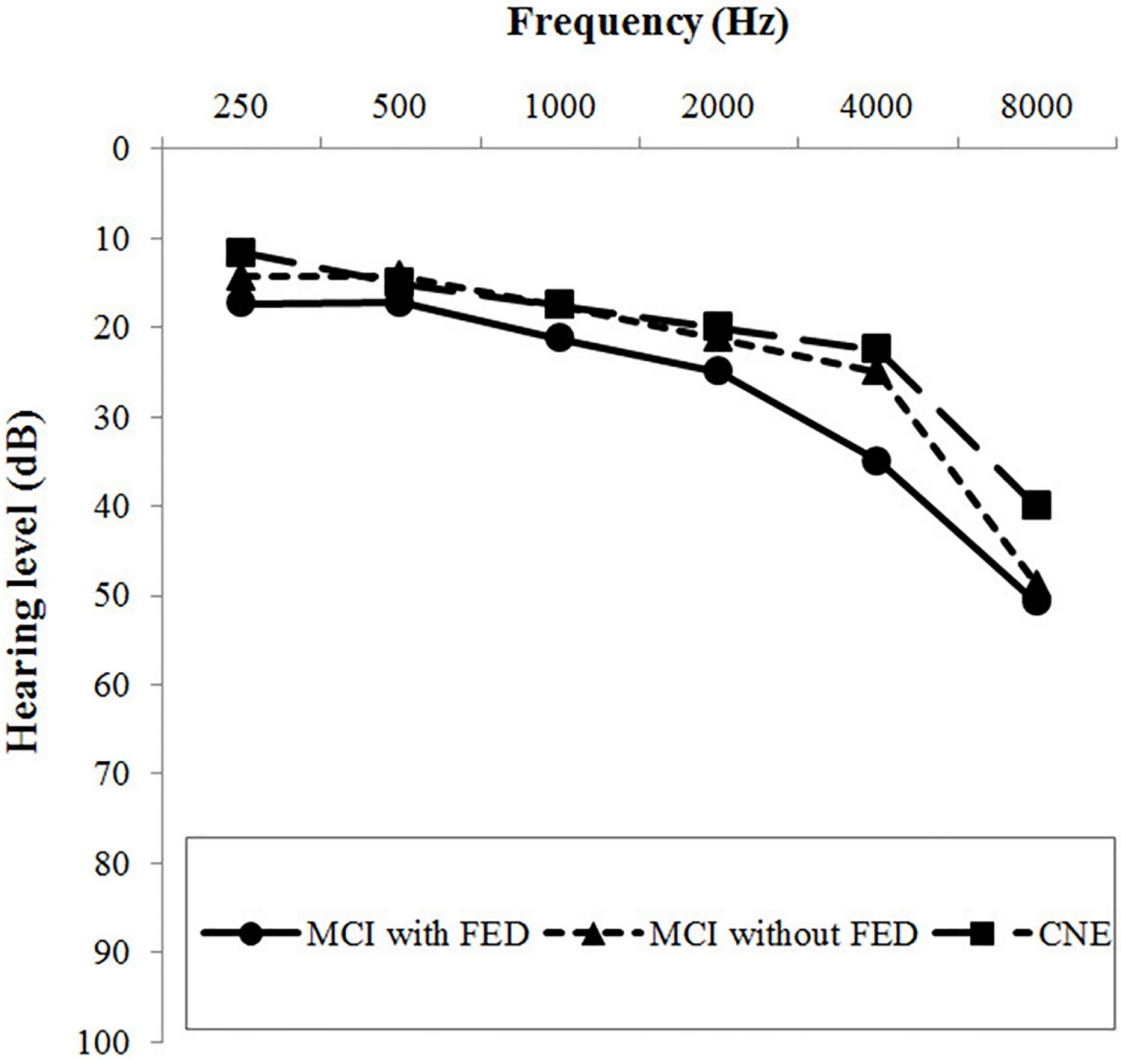
Means or medians of the binaural average of hearing thresholds at each frequency in mild cognitive impairment (MCI) with frontal-executive dysfunction (FED), MCI without FED, and cognitively normal elderly (CNE) groups.

All participants underwent pure-tone audiometry, speech audiometry, tympanometry, and speech-in-noise perception tests. Air and bone conduction thresholds were measured with a clinical pure-tone audiometer (GSI 61; Grason-Stadler, Eden Prairie, MN, USA). We calculated the PTAs at 0.5, 1, 2, and 4 kHz for each ear. We measured the speech reception threshold, speech discrimination score, and most comfortable loudness level for each ear. Speech-in-noise perception was measured via sentence recognition tests with speech-spectrum and multi-talker babble background noise at a signal-to-noise ratio of -5 dB. The means or medians of the binaural average of the pure-tone audiometric thresholds at each frequency are shown in pure-tone audiograms for each group (Fig 1).

#### Neuropsychological assessments

All participants underwent a standardized neuropsychological test, the Seoul Neuropsychological Screening Battery (SNSB) [20]. The SNSB evaluates five cognitive domains: attention, language, visuospatial function, visual and verbal memory, and frontal-executive function. Attention was assessed using forward and backward digit span tests. Language was examined using the Korean version of the Boston Naming Test (K-BNT) [23]. Visuospatial function and visual memory were measured using the Rey Complex Figure Test (RCFT), which involves copying, immediate recall, 20-minute delayed recall, and recognition. Verbal memory was evaluated using the SVLT, which involves three trials: immediate recall, 20-minute delayed recall, and recognition of 12 words. Frontal-executive function was determined using the COWAT, which assesses semantic and phonemic verbal fluency, and the K-CWST, which involves word and color reading of 112 items. On the K-CWST, participants are required to read 112 color words printed in incongruent colored ink in 120 seconds (i.e., word reading task) and subsequently, to name the ink color of the color words, which does not correspond to the semantic meaning of the color words, in 120 seconds (i.e., color reading task). The interference score of the K-CWST is calculated by subtracting the time-per-item of the word reading from the time-per-item of the color reading. We administered the Short version of the Geriatric Depression Scale (SGDS) [24] consisting of 15 questions to assess participants’ levels of depression.

#### Assessment of self-perceived hearing handicap

All participants completed the Korean version of the Hearing Handicap Inventory for the Elderly (K-HHIE) [25,26]. Self-perceived hearing handicap was supposed to be assessed using the Hearing Handicap Inventory for Adults (HHIA) in participants below 65 years old; however, because no standardized Korean version of the HHIA is available, all participants aged 55 to 80 years underwent the K-HHIE in this study. In addition, the reliability of the K-HHIE in all respondents aged over 20 years is the same as that in respondents aged over 65 years (i.e., Cronbach’s alpha coefficient = 0.95), suggesting that the K-HHIE can be adapted to all age groups [26]. The K-HHIE is composed of 25 items; among these items, 13 involve emotional aspects (e.g., “Does a hearing problem make you irritable?”), and 12 involve social/situational aspects (e.g., “Does a hearing problem cause you to avoid groups of people?”). Respondents are asked to select “yes,” “sometimes,” or “no” for each item (4, 2, or 0 points, respectively), and the responses are combined to generate emotional, social/situational, and total scores. Scores range from a minimum of 0 to a maximum of 48, 52, and 100 for social/situational, emotional, and total scores, respectively. High scores indicate a significant perception of hearing handicap by respondents.

### Statistical analysis

All continuous variables were first tested for normality using the Shapiro-Wilk test. Continuous variables not normally distributed, presented as medians and interquartile ranges, were analyzed using the Kruskal-Wallis test. However, the continuous variables normally distributed, presented as mean and SD, were analyzed using the one-way analysis of variance. Second, because normality was rejected for the hearing handicap scores, nonparametric analysis of covariance based on Quade’s test in SPSS was used to compare the hearing handicap scores among groups after adjusting for age, years of education, and depression levels. Post-hoc analyses were conducted using pairwise comparisons with Bonferroni corrections to examine the differences among groups. Additionally, multiple regression analysis using the backward elimination approach was performed in order to determine which independent variables, including cognitive impairment, age, years of education, and depression levels, were predictors of the K-HHIE total scores for the entire sample. Third, for nonparametric partial correlation, Spearman rank correlation coefficients (rho) were computed by controlling for depression levels in each group to examine the correlations of self-perceived hearing handicap with demographic, audiometric, and neuropsychological variables. Alpha levels were set at *P* = 0.05, and statistical analyses were carried out using SPSS 23.0 software.

## Results

### Comparison of K-HHIE scores between the MCI and CNE groups

After controlling for age, years of education, and depression levels, we found no significant main effects of group on social/situational (*F*_1,83_ = 2.590, *P* = 0.111), emotional (*F*_1,83_ = 1.494, *P* = 0.225), and total scores (*F*_1,83_ = 2.873, *P* = 0.094) on the K-HHIE. In addition, multiple regression analysis was performed using the backward elimination approach in order to determine which independent variables, including cognitive impairment (MCI, CNE), age, years of education, and depression levels, were predictors of the K-HHIE total scores for the entire sample. In the final model (*F*_2,82_ = 20.133, *P* < 0.001, adjusted R^2^ = 0.313), depression levels (*B* = 1.097, *SE B* = 0.204, β = 0.497, *P* < 0.001) and years of education (*B* = −0.364, *SE B* = 0.168, β = −0.201, *P* = 0.033) remained significant while age (*P* = 0.917) and cognitive impairment (*P* = 0.273) were eliminated from the analysis. Simple linear regression analysis confirmed that the years of education and depression levels were significantly correlated with the K-HHIE total scores, respectively (correlation coefficient = −0.328, estimate of slope = −0.640, *P* < 0.001; correlation coefficient = 0.503, estimate of slope = 1.132, *P* < 0.001).

### Comparison of K-HHIE scores among the MCI with FED, MCI without FED, and CNE groups

**Fig 2:**
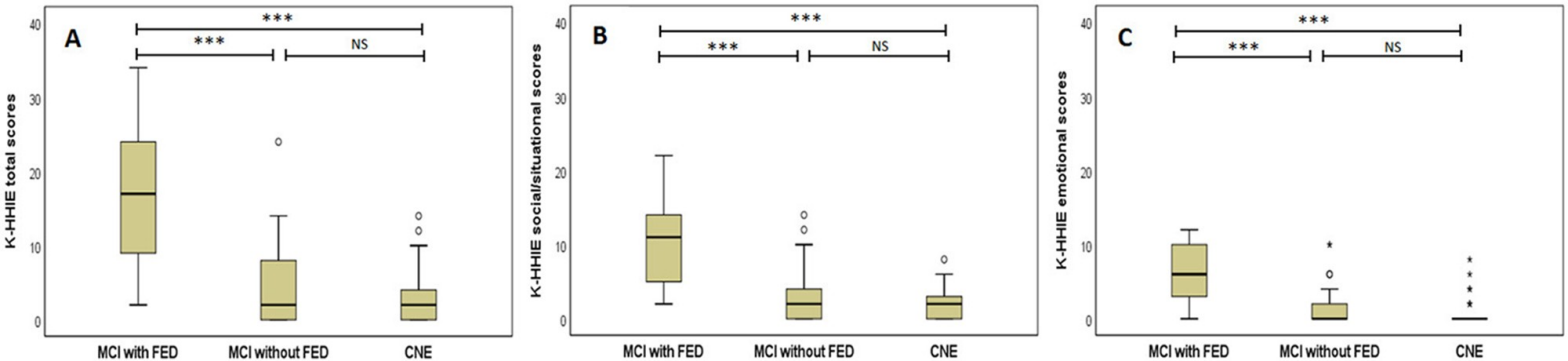
Comparisons of the total (A), social/situational (B), and emotional (C) scores of the Korean version of the Hearing Handicap Inventory for the Elderly (K-HHIE) among the MCI with FED, MCI without FED, and CNE groups. ***P < 0.001. NS, not significant.

After controlling for age, years of education, and depression levels, we found significant main effects of group on social/situational (*F*_2,82_ = 9.576, *P* < 0.001), emotional (*F*_2,82_ = 12.243, *P* < 0.001), and total scores (*F*_2,82_ = 10.889, *P* < 0.001) on the K-HHIE. Post hoc analysis revealed that the MCI with FED group scored significantly higher than did both the MCI without FED (*P* < 0.001) and the CNE (*P* < 0.001) groups on the social/situational section. In addition, the MCI with FED group scored significantly higher than did the MCI without FED (*P* < 0.001) and the CNE (*P* < 0.001) groups on the emotional section. Moreover, the MCI with FED group scored significantly higher in total scores than did the MCI without FED (*P* < 0.001) and the CNE (*P* < 0.001) groups. No significant differences were found between the MCI without FED and the CNE groups on the social/situational, emotional, and total scores on the K-HHIE (Fig 2).

In addition, we also performed multiple regression analysis using the backward elimination approach in order to determine which independent variables, including cognitive impairment (MCI with FED, MCI without FED, and CNE), age, years of education, and depression levels, were predictors of the K-HHIE total scores for the entire sample. Dummy coding was conducted for cognitive impairment with the MCI with FED group as the reference group. In the final model (*F*_3,81_ = 32.427, *P* < 0.001, adjusted R^2^ = 0.529), CNE (dummy variable) (*B* = −10.939, *SE B* = 1.882, β = −0.676, *P* < 0.001), MCI without FED (dummy variable) (*B* = −11.148, *SE B* = 1.741, β = −0.661, *P* < 0.001), and depression levels (*B* = 0.799, *SE B* = 0.195, β = 0.362, *P* < 0.001) remained significant while age (*P* = 0.537) and years of education (*P* = 0.143) were eliminated from the analysis.

### Partial correlations between K-HHIE total scores and demographic, audiometric, or neuropsychological variables in each group

**Fig 3:**
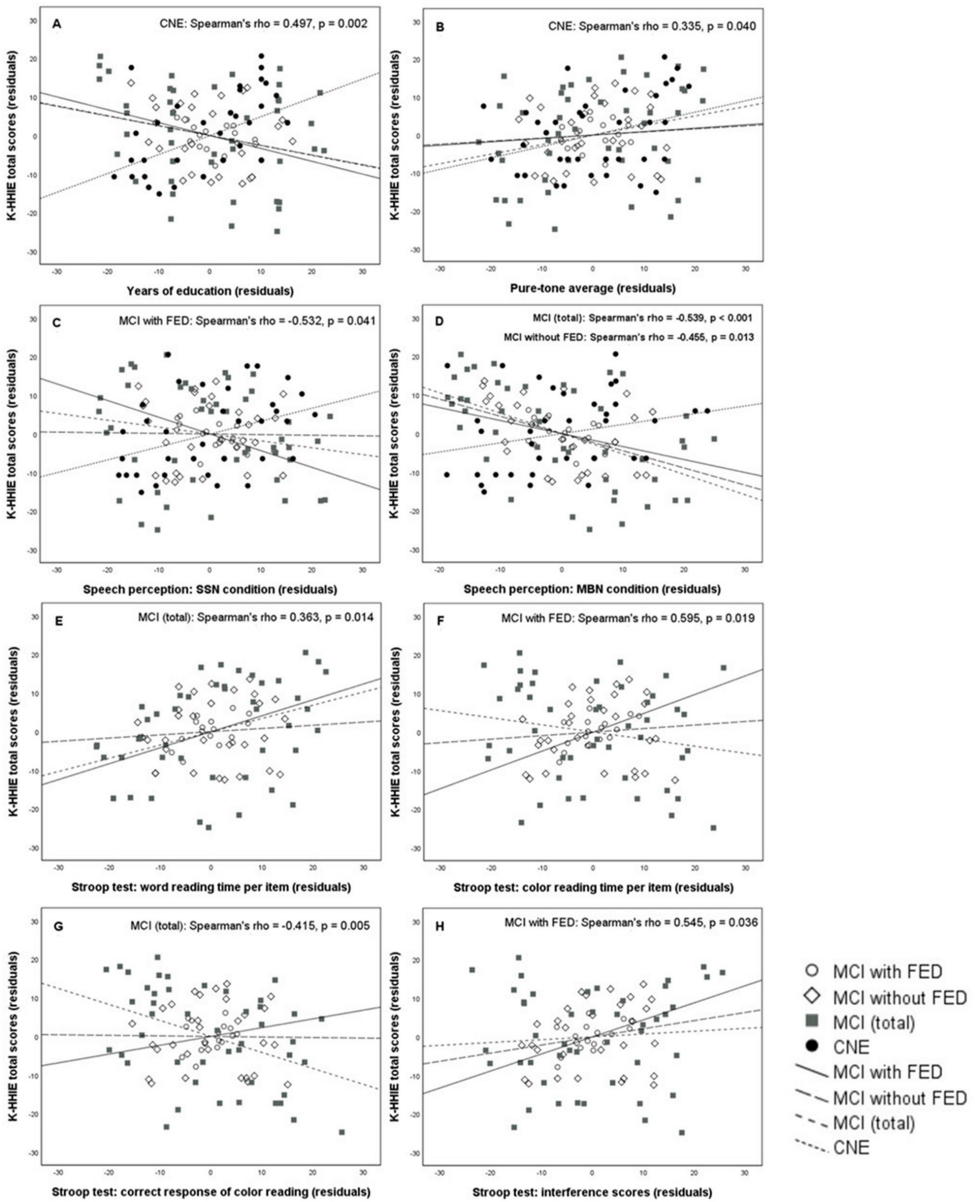
Scatter plots show the partial correlations between the K-HHIE total score and each of the eight variables in the MCI with FED, MCI without FED, total MCI, and CNE groups. Variables: years of education (A); pure-tone average (B); sentence recognition scores in speech-spectrum noise (C) and multi-talker babble noise (D) conditions; and word reading time per item (E), color reading time per item (F), number of correct responses of color reading (G), and interference scores (H) in the K-CWST. For all panels, a group showing statistically significant correlation is displayed on the top right corner. For example, in the panel A, only the CNE group shows a statistically significant correlation between the K-HHIE total score and years of education.

After controlling for depression levels in the MCI with FED group, we found significant correlations between the K-HHIE total score and the sentence recognition score in the speech-spectrum noise condition (Spearman’s rho = −0.532, *P* = 0.041), color reading time per item (Spearman’s rho = 0.595, *P* = 0.019), or interference scores (Spearman’s rho = 0.545, *P* = 0.036) in the K-CWST. In the MCI without FED group, we found a significant correlation between the K-HHIE total score and the sentence recognition score in the multi-talker babble noise condition (Spearman’s rho = −0.455, *P* = 0.013). In the total MCI group, we found significant correlations between the K-HHIE total score and the sentence recognition score in the multi-talker babble noise condition (Spearman’s rho = −0.539, *P* < 0.001), word reading time per item (Spearman’s rho = 0.363, *P* = 0.014), or color reading scores (Spearman’s rho = −0.415, *P* = 0.005) in the K-CWST. In the CNE group, we found significant correlations of the K-HHIE total score with the PTA (Spearman’s rho = 0.335, *P* = 0.040) or years of education (Spearman’s rho = 0.497, *P* = 0.002) (Table 2). Scatter plots of the significant partial correlations are shown in Fig 3.

**Table 2:** Partial correlation coefficients of the K-HHIE total score with demographic, audiometric, and neuropsychological variables adjusted by depression levels in each group.

	MCI			CNE^†^ (n = 39)
	MCI with FED (n = 16)	MCI without FED (n = 30)	MCI (total) (n = 46)	
Demographics				
Age	0.397[−0.036, 0.765]	−0.026[−0.385, 0.350]	−0.017[−0.316, 0.291]	−0.223[−0.529, 0.099]
Education	−0.408[−0.683, 0.026]	−0.251[−0.569, 0.119]	−0.267[−0.548, 0.076]	0.497**[0.143, 0.766]
Audiometric tests				
PTAs	0.105[−0.246, 0.494]	0.083[−0.264, 0.437]	0.260[−0.030, 0.548]	0.335*[0.021, 0.602]
SDS	−0.013[−0.504, 0.408]	0.041[−0.344, 0.405]	−0.029[−0.341, 0.271]	−0.102[−0.446, 0.332]
Speech-in-noise perception				
SSN condition	−0.532*[−0.842, −0.113]	−0.016[−0.380, 0.396]	−0.187[−0.492, 0.133]	0.261[−0.082, 0.578]
MBN condition	−0.378[−0.767, 0.228]	−0.455*[−0.727, −0.152]	−0.539***[−0.699, −0.365]	0.250[−0.075, 0.567]
Neuropsychological tests^‡^				
K-MMSE	−0.160[−0.494, 0.250]	−0.100[−0.422, 0.231]	−0.267[−0.503, 0.032]	0.179[−0.096, 0.413]
Attention: Digit Span				
Forward	−0.040[−0.523, 0.421]	−0.239[−0.561, 0.111]	−0.058[−0.310, 0.229]	0.002[−0.331, 0.332]
Backward	−0.042[−0.464, 0.486]	0.116[−0.275, 0.451]	−0.068[−0.380, 0.224]	0.197[−0.131, 0.525]
Language: K-BNT	0.048[−0.580, 0.538]	−0.046[−0.383, 0.324]	−0.100[−0.385, 0.158]	-
Visuospatial function				
RCFT copy	−0.420[−0.722, 0.181]	−0.048[−0.364, 0.268]	−0.139[−0.427, 0.130]	-
RCFT copy time	−0.341[−0.809, 0.160]	0.171[−0.261, 0.588]	−0.056[−0.352, 0.238]	-
Verbal memory: SVLT				
Immediate recall	−0.189[−0.756, 0.516]	0.251[−0.089, 0.527]	−0.020[−0.300, 0.255]	0.075[−0.210, 0.376]
Delayed recall	−0.086[−0.650, 0.588]	0.274[−0.092, 0.594]	0.034[−0.260, 0.323]	−0.056[−0.339, 0.235]
Recognition	0.360[−0.194, 0.871]	0.016[−0.332, 0.371]	−0.094[−0.397, 0.219]	−0.044[−0.318, 0.224]
Visual memory: RCFT				
Immediate recall	−0.133[−0.502, 0.284]	0.136[−0.273, 0.489]	−0.031[−0.343, 0.276]	-
Delayed recall	−0.113[−0.433, 0.212]	0.135[−0.244, 0.433]	−0.044[−0.357, 0.284]	-
Recognition	0.004[−0.421, 0.429]	0.064[−0.282, 0.389]	0.062[−0.258, 0.380]	-
Frontal-executive function				
COWAT: semantic				
Animals	−0.419[−0.723, 0.029]	0.217[−0.158, 0.599]	−0.127[−0.394, 0.150]	0.052[−0.300, 0.385]
Supermarket	0.275[−0.205, 0.621]	−0.339[−0.664, 0.025]	−0.271[−0.503, 0.013]	0.025[−0.276, 0.343]
COWAT: phonemic, total	0.060[−0.499, 0.638]	−0.207[−0.565, 0.203]	−0.227[−0.484, 0.051]	0.245[−0.058, 0.492]
K-CWST: word reading	−0.508[−0.830, 0.012]	0.083[−0.317, 0.455]	−0.328[−0.553, 0.052]	-
Time per item	0.498[−0.090, 0.805]	0.085[−0.247, 0.426]	0.363*[0.119, 0.578]	-
K-CWST: color reading	0.214[−0.320, 0.671]	−0.014[−0.374, 0.365]	−0.415**[−0.641, −0.140]	-
Time per item	0.595*[0.056, 0.907]	0.092[−0.273, 0.505]	−0.192[−0.502, 0.135]	-
K-CWST: interference score	0.545*[0.100, 0.834]	0.213[−0.176, 0.585]	0.076[−0.287, 0.423]	-

The lower and upper limits of the 95% confidence intervals using the bootstrap method are shown in square brackets. Only the correlation between the K-HHIE total score and the sentence recognition score in the multi-talker babble noise condition in the total MCI group remained significant after applying a Bonferroni correction for multiple comparisons (*P* < 0.001).

*P < 0.05,

**P < 0.01,

***P < 0.001.

^†^Among neuropsychological tests, only the K-MMSE, SVLT, COWAT, and digit span tests were conducted in the CNE group.

^‡^The neuropsychological test results were converted into age- and education-adjusted z-scores based on the published normative data.

Abbreviations: K-HHIE, Korean version of Hearing Handicap Inventory for the Elderly; MCI, mild cognitive impairment; FED, frontal-executive dysfunction; CNE, cognitively normal elderly; PTAs, pure-tone averages; SDS, speech discrimination score; SSN, speech-spectrum noise; MBN, multi-talker babble noise; K-MMSE, Korean version of Mini-Mental State Examination; K-BNT, Korean version of Boston Naming Test; RCFT, Rey Complex Figure Test; SVLT, Seoul Verbal Learning Test; COWAT, Controlled Oral Word Association Test; K-CWST, Korean version of Color Word Stroop Test.

## Discussion

It is increasingly agreed that cognitive factors and audiological factors are associated with self-perceived hearing handicap, especially in old adults, suggesting that old adults with cognitive impairments may have more hearing-related difficulties than their hearing threshold-matched peers with normal cognitive function. As old adults with hearing handicap experience emotional distress and restrictions on social engagement in everyday life, which ultimately reduce their quality of life [2,27], it is important to study which factors adversely impact hearing handicap and to identify which groups are more likely to experience hearing difficulties in everyday life. In this study, the self-reported hearing handicap in MCI patients was compared with that in hearing threshold-matched CNE subjects. The factors associated with hearing handicap in each group were also examined. Four points in our findings bear further discussion.

First, MCI patients with FED are more likely to experience hearing difficulties in their daily lives compared with those without FED or cognitively normal old adults. Considering the relationship between working memory capacity and executive functioning [28], the findings in the present study are somewhat inconsistent with those in a previous study [12], which indicates that old adults with high working memory capacity reported more hearing difficulties. Because working memory is one of the core executive functions, along with response inhibition, interference control, and cognitive flexibility [29], working memory capacity and frontal-executive function tasks share a common underlying executive attention component [28]. On this basis, we interpret the present results with regard to working memory capacity although working memory capacity was not directly investigated as a diagnostic criterion for FED. Prior studies have reported the positive association between working memory capacity and reported hearing difficulties. However, we found the negative correlation between executive function scores and self-reported hearing handicap in patients with MCI. In fact, it is quite common to experience hearing difficulties when executive functions are disrupted. For example, one listener with executive dysfunction may report more difficulties than another listener with a similar degree of hearing loss but intact executive functioning in response to the following question: “Does a hearing problem cause you difficulty when attending a party?” Having conversations with people at a party requires both good cognitive abilities and hearing to focus selectively on target speech while filtering out other conversations [30,31]. Therefore, deficits in frontal-executive function (e.g., working memory, behavioral inhibition, and selective attention) can increase the social-emotional consequences of hearing impairments.

In the present study, among measures on frontal-executive function, variables related to the K-CWST were specifically correlated with hearing handicap scores in the MCI groups. In the MCI with FED group, hearing handicap scores were positively correlated with the color reading time per item and interference score, which is calculated by subtracting the time per item of the word reading from the time per item of the color reading. Moreover, in the total MCI group, hearing handicap scores were positively correlated with the word reading time per item and were negatively correlated with the color reading scores. The Stroop test mainly assesses the ability to inhibit cognitive interference and the processing speed by measuring the accuracy and speed of performance [32]. Because participants are required to perform a less automated task (i.e., naming the ink color) while inhibiting the interference arising from a more automated task (i.e., reading the written word), the Stroop effect increases on the color reading task [17]. Therefore, our results indicate that the more vulnerable the patients are to cognitive interference, the more hearing difficulties they report in their daily lives. Furthermore, our findings also indicate that slowing in processing speed, reflected by increased reading time per item on the Stroop test, is associated with increased hearing handicap in the patients with MCI.

Second, speech perception performance is significantly correlated with self-perceived hearing handicap in patients with MCI. Specifically, hearing handicap scores were negatively correlated with the sentence recognition scores in the multi-talker babble noise condition in the MCI without FED and total MCI groups and in the speech-spectrum noise condition in the MCI with FED group. However, it should be noted that only the correlation between the K-HHIE total score and the sentence recognition score in the multi-talker babble noise condition in the total MCI group remained significant after applying a Bonferroni correction for multiple comparisons. Several previous studies indicate an association between self-perceived hearing handicap and outcomes on speech-in-noise measures [4–8]. The findings suggest that deficits in speech-in-noise perception tests reflect subjective hearing difficulties in daily life and that multi-talker babble noise conditions better reflect noisy listening situations than do speech-spectrum noise conditions in everyday life. It is well known that speech perception is cognitively more demanding when it is masked by interfering speech, such as multi-talker babble noise (i.e., informational masking), than meaningless noise, such as speech-spectrum noise (i.e., energetic masking) [30,31,33]. Therefore, patients with MCI are more likely to have difficulties in the presence of informational masking than energetic masking. Interestingly and unexpectedly, no such correlations were found in the CNE group. The findings may suggest that other audiological factors (e.g., peripheral hearing sensitivity), rather than speech-in-noise perception or cognitive factors, have great effects on subjective hearing difficulties in cognitively normal old adults.

Third, peripheral hearing sensitivity explains little of the variance in MCI patients’ self-reported hearing handicap. The degree of peripheral hearing loss was not one of the factors correlated with subjective hearing difficulties in the MCI patients. In the CNE group, we found a relatively low correlation between hearing sensitivity and self-perceived hearing handicap. In addition, although numerous studies have shown that peripheral hearing sensitivity is correlated with self-perceived hearing handicap in old listeners, the correlations are only weak to moderate [1,10]. Moreover, audiometric measures only explain less than half of the variance in hearing handicap [11]. These findings indicate that hearing impairment does not parallel the self-perception of hearing handicap in everyday life in old adults. Especially, in old adults with cognitive impairment, cognitive function and/or speech-in-noise perception performance are more likely to be associated with hearing handicap.

The fourth discussion point is that we found a significant positive correlation between hearing handicap scores and years of education in the CNE group. Although no such correlations were found in the MCI groups, old adults with higher education reported greater hearing handicap than those with lower education in the CNE group. The results are somewhat consistent with those in a previous study, which demonstrates that less than 12 years of education is an associated factor for less self-perceived hearing handicap [34]. The association between higher educational level and more hearing difficulties could be because cognitively normal old adults with high education tend to have greater demands on hearing and communication abilities in their lifestyles, thus better identifying their difficulties in real life. In contrast, in the cognitively impaired old adults, cognitive factors other than the educational level may highly affect self-perceived hearing handicap.

Interestingly, multiple regression analysis for the entire sample revealed that years of education and depression levels were possible predictors of the hearing handicap scores, but the correlation between hearing handicap and years of education was negative. This negative correlation for the entire sample was in the opposite direction from that for the CNE group. This result could suggest that educational level may affect hearing handicap differently in cognitively normal old adults and in cognitively impaired old patients. As shown in Fig 3A, inconsistent with the positive correlation observed in the CNE group, the correlations between years of education and hearing handicap were all negative in the MCI groups although none of them was statistically significant. However, very few studies have investigated the effect of the educational level on hearing handicap both in the cognitively normal and in the cognitively impaired elderly groups. Therefore, further studies are needed to confirm the present findings.

Finally, multiple regression analysis for the entire sample confirmed that depression level was also a significant positive predictor of hearing handicap scores. That is, the more that older listeners showed symptoms of depression, the more they had hearing difficulties. It is well known that depression symptoms adversely impact cognitive function in elderly people [35]. Symptoms of depression may be associated with hearing handicap through a casual pathway mediated by cognitive dysfunction and increased cognitive load in daily life. Although some previous studies also indicated that depression signs were related to self-perceived hearing handicap [36–38], further research on the effect of depression on hearing handicap is necessary.

Notably, this study suggests that MCI patients with FED experience more hearing difficulties than do their peers with normal cognitive function or MCI patients without FED in daily life. Cognitive function, especially frontal-executive function and speech-in-noise perception performance, is correlated with self-perceived hearing handicap in elderly patients with MCI. This study indicates that, in clinical practice, the self-assessment of hearing handicap, in addition to audiometric assessments, needs to be conducted in order to evaluate each individual’s hearing difficulties in daily life. If old adults report more hearing problems than do their hearing threshold-matched peers, they may have cognitive impairments, especially in frontal-executive function. Therefore, those old adults should be considered for assessment and intervention on neuropsychological function. In audiological settings, clinicians can provide proper intervention services to these patients by early referral to neuropsychology and/or aural rehabilitation specialists.

Despite the strengths of our study, it also has some limitations. First, in the present study, we controlled for hearing thresholds, age, years of education, and depression levels to investigate the effect of cognitive function on self-perceived hearing handicap. Although participants’ depressive symptoms were evaluated and adjusted in this study and the relations between depression and self-perceived hearing handicap were indicated in the literature [36–38], other psychological symptoms besides depression (e.g., anxiety and apathy) could affect hearing handicap. Therefore, further studies are needed to conduct a comprehensive psychological assessment and to administer formal diagnostic criteria such as the Structured Clinical Interview for Diagnostic and Statistical Manual of Mental Disorders (SCID) [39] for the assessment of psychiatric diagnoses. Moreover, in this study, we could not control for chronic health conditions (e.g., hypertension and diabetes) in the participants. To date, it is still unclear which potential risk factors (e.g., general health status and personality traits) [1,40] may affect degrees of self-perceived hearing handicap; therefore, further studies with systematically control for potential confounders are needed to better understand the effect of cognitive function on hearing handicap. Second, although the HHIE is the most commonly used tool for evaluation of hearing handicap in the elderly [3], the HHIE alone might not fully evaluate the social/situational and emotional consequences of hearing impairment. Third, because this study was cross-sectional in design, it would be greatly preferable to obtain longitudinal data to assess long-term relations between cognitive function and hearing handicap. Lastly, further studies with a large sample size would be more representative of the MCI population and thus would be useful to confirm the current findings.

## Supporting information

S1 Table Means or medians of neuropsychological test scores for individual tests in each group. (DOCX)
