# Supplementary material for: Effects of frontal-executive dysfunction on self-perceived hearing handicap in the elderly with mild cognitive impairment
Source: PLoS One. 2019 Mar 6;14(3):e0210014. doi: 10.1371/journal.pone.0210014 (PMC6402624; doi:10.1371/journal.pone.0210014)
Supplement: S1 Table — (DOCX) [file pone.0210014.s001.docx]

[Supporting Information]

| **S1 Table. Means or medians of neuropsychological test scores for individual tests in each group.** | | | | | | |
| --- | --- | --- | --- | --- | --- | --- |
|  | Raw scores | | | Age- and education-adjusted Z scores^†^ | | |
|  | MCI  with FED | MCI  without FED | CNE^‡^ | MCI  with FED | MCI  without FED | CNE^‡^ |
| SGDS | 4.00 (2.25−11.00) | 3.50 (2.00−8.00) | 1.00 (0.00−2.00) | - | - | - |
| K-MMSE | 25.00 (24.00−26.00) | 26.00 (24.75−29.00) | 29.00 (28.00−30.00) | -1.566 ± 1.322 | -0.613 ± 1.323 | 0.606 ± 0.809 |
| Attention: Digit span |  |  |  |  |  |  |
| Forward | 6.00 (5.00−7.75) | 6.00 (5.00−7.00) | 8.00 (6.00−9.00) | 0.311 ± 1.323 | 0.179 ± 1.156 | 1.284 ± 0.800 |
| Backward | 3.00 (2.00−3.00) | 3.00 (3.00−4.00) | 4.00 (4.00−5.00) | -0.848 ± 0.834 | -0.421 ± 0.737 | 1.053 ± 1.291 |
| Language: K-BNT | 39.81 ± 6.22 | 41.90 ± 8.12 | - | -1.181 ± 0.882 | -0.735 ± 1.250 | - |
| Visuospatial function |  |  |  |  |  |  |
| RCFT copy | 31.00 (21.25−36.00) | 32.50 (29.25−36.00) | - | -0.540 ± 1.792 | -0.009 ± 1.077 | - |
| RCFT copy time (in sec) | 285.62 ± 147.12 | 269.43 ± 122.69 | - | -0.750 ± 1.622 | -0.481 ± 1.109 | - |
| Verbal memory: SVLT |  |  |  |  |  |  |
| Immediate recall | 14.50 (9.75−17.00) | 18.00 (13.75−21.00) | 18.00 (17.00−23.00) | -1.298 ± 0.976 | -0.379 ± 1.151 | 0.228 ± 0.982 |
| Delayed recall | 3.00 (1.00−5.00) | 4.00 (3.00−6.25) | 6.00 (5.00−8.00) | -1.369 ± 0.918 | -0.828 ± 1.463 | -0.001 ± 0.835 |
| Recognition | 18.00 (17.25−20.00) | 20.00 (19.00−21.25) | 21.00 (20.00−22.00) | -1.442 ± 2.047 | -0.284 ± 0.915 | 0.226 ± 0.922 |
| Visual memory: RCFT |  |  |  |  |  |  |
| Immediate recall | 8.71 ± 6.53 | 9.56 ± 5.37 | - | -0.850 ± 1.059 | -0.705 ± 0.847 | - |
| Delayed recall | 9.03 ± 7.04 | 10.01 ± 5.82 | - | -0.898 ± 1.218 | -0.626 ± 0.902 | - |
| Recognition | 19.00 (18.00−21.00) | 20.00 (18.00−21.00) | - | -0.547 ± 1.519 | -0.302 ± 0.994 | - |
| Frontal-executive function |  |  |  |  |  |  |
| COWAT: semantic |  |  |  |  |  |  |
| Animals | 10.37 ± 3.03 | 12.63 ± 3.54 | 18.07 ± 4.38 | -1.229 ± 0.635 | -0.571 ± 1.006 | 0.735 ± 1.421 |
| Supermarket | 11.43 ± 3.57 | 14.60 ± 4.72 | 20.48 ± 5.98 | -0.948 ± 0.846 | -0.323 ± 0.891 | 0.560 ± 1.033 |
| COWAT: phonemic, total | 11.81 ± 5.76 | 17.43 ± 9.19 | 28.17 ± 12.21 | -1.236 ± 0.707 | -0.576 ± 1.082 | 0.534 ± 1.326 |
| K-CWST: word reading | 111.00 (97.75−112.00) | 112.00 (111.00−112.00) | - | -0.367 ± 1.513 | 0.195 ± 0.503 | - |
| time per item (in sec) | 0.90 (0.71−1.18) | 0.65 (0.60−0.89) | - | -2.838 ± 7.662 | 0.156 ± 0.509 | - |
| K-CWST: color reading | 50.00 (35.00−59.75) | 84.00 (73.50−94.00) | - | -1.946 ± 0.741 | 0.048 ± 0.830 | - |
| time per item (in sec) | 2.07 (1.75−2.68) | 1.43 (1.27−1.62) | - | -1.684 ± 1.455 | 0.002 ± 0.708 | - |
| K-CWST: interference score | 1.06 (0.54−1.64) | 0.74 (0.57−0.82) | - | -0.235 ± 4.522 | -0.090 ± 0.706 | - |

Parametric data are presented as mean ± standard deviation and nonparametric data as median (interquartile range). ^†^The neuropsychological test results except for the SGDS were converted into age- and education-adjusted z-scores based on the published normative data. ^‡^Among neuropsychological tests, only the SGDS, K-MMSE, SVLT, COWAT, and digit span tests were conducted for the CNE group.

Abbreviations: MCI, mild cognitive impairment; FED, frontal-executive dysfunction; CNE, cognitively normal elderly; SGDS, Short version of Geriatric Depression Scale; K-MMSE, Korean version of Mini-Mental State Examination; K-BNT, Korean version of Boston Naming Test; RCFT, Rey Complex Figure Test; SVLT, Seoul Verbal Learning Test; COWAT, Controlled Oral Word Association Test; K-CWST, Korean version of Color Word Stroop Test.
